# Defibrillation pad placement accuracy among Advanced Life Support instructors: A manikin-based observational study examining experience, self-evaluation, and actual performance

**DOI:** 10.1016/j.resplu.2025.100886

**Published:** 2025-02-01

**Authors:** Dennie Wulterkens, Freek Coumou, Cornelis Slagt, Reinier A. Waalewijn, Lars Mommers

**Affiliations:** a Institute of Quality Medical Training Nieuwegein the Netherlands; b Helicopter Emergency Medical Service Lifeliner 3 Nijmegen the Netherlands; c Department of Anaesthesiology Pain and Palliative Medicine Radboud University Medical Centre Nijmegen the Netherlands; d Department of Cardiology Gelre Hospitals the Netherlands and St. Antonius Hospital Nieuwegein the Netherlands; e Department of Anaesthesiology and Pain Medicine Maastricht University Medical Centre Maastricht the Netherlands; f Department of Simulation in Healthcare Maastricht University Medical Centre Maastricht the Netherlands

**Keywords:** Defibrillation, Ventricular fibrillation, Simulation, Cardiac arrest, Teaching, Pads placement

## Abstract

**Background:**

Ventricular fibrillation is common in patients with out-of-hospital cardiac arrest. Early and effective defibrillation is important for their survival. Effective defibrillation depends highly on correct positioning of the defibrillation pads. Teaching this correctly by ALS instructors is therefore crucial.

**Methods:**

Fifty certified advanced life support instructors were recruited from a large training institute. Participants were asked to place defibrillation pads on an anatomically and real-weight (90 kg) manikin. Primary outcome was the placement of defibrillation pads placed in the sternal-apical and anterior-posterior positions. Secondary outcomes were performance self-assessment, defibrillation experience, self-perceived competence and self-efficacy in teaching defibrillation. These measures were evaluated using an 11-point Likert scale.

**Results:**

A total of 31 medical doctors and 19 registered nurses were enrolled in this study. Defibrillation pads were placed (mean ± SD) 42 ± 21 mm, 38 ± 23 mm, 35 ± 19 mm and 61 ± 48 mm from the reference point for the sternal, apical, anterior and posterior pads respectively, resulting in a respectively correct placement of 18%, 20%, 32% and 28%. The average number of correctly applied pads per instructor was 0.98 ± 0.74 out of four.

Self-assessment of defibrillation pads placed by the participants were 8.56 ± 1.33 and 7.88 ± 1.64 for the sternal-apical and anterior-posterior positions respectively. Personal defibrillation experience showed that the majority had applied over 20 standard defibrillations. Experience with anterior-posterior pad placement was less and experience with bi-axillary and double sequential external defibrillation positions were absent in most participants. Self-perceived competence for the sternal-apical, anterior-posterior, bi-axillary and dual external synchronized positions were 8.68 ± 1.06, 8.08 ± 1.37, 5.57 ± 2.95 and 5.11 ± 2.67 respectively. Self-efficacy score for teaching defibrillation was 8.59 ± 0.81. No association was found between the number of correctly applied pads and any of the participants’ variables.

**Conclusion:**

This study corroborates and expands upon existing knowledge regarding the challenges of defibrillator pad placement, revealing substantial variation in placement accuracy among instructors. Our novel analysis of pad angles and anterior-posterior analysis demonstrates that a significant portion of pads are incorrectly placed. These findings highlight the need for standardized approaches and improved training methodologies in defibrillator pad placement.

## Introduction

Ventricular fibrillation is common in out-of-hospital cardiac arrest.^1^ Given the rapid decline in survival, early and effective treatment is important for a favourable outcome. Effective defibrillation is emphasized in the well-established Advanced Life Support (ALS) resuscitation guidelines by the European Resuscitation Council (ERC).^2^ It is of the utmost importance that ALS instructors teach the correct pad positionings.

In order for defibrillation the be effective, sufficient energy (*electrical current)* has to pass through the myocardium (*correct vector*) to result in a *transcardiac current*. Only a minority (4%) of the *transthoracic current* contributes to the transcardiac current as the majority shunts away through other pathways (e.g. thoracic cage, lungs).^3^ The transthoracic current is determined by multiple variables, including the shape of the thorax, pulmonary volume, body weight as well as the surface area and position of the defibrillation electrodes.^4^ Although modern defibrillators can compensate for transthoracic impedance (*impedance-compensated biphasic waveform delivery*), they cannot compensate the electrical vector and therefore correct defibrillator pad application remains essential.^4^

The current ERC guideline states that defibrillation pads should be placed in the conventional ‘sternal-apical’ (SA) position. The sternal pad should be placed ‘to the right of the sternum, below the clavicle’ and the apical pad should be placed ‘in the left mid-axillary line, approximately level with the V6 ECG electrode’, making sure this place is free of breast tissue and sufficiently lateral.^2^ Alternative pad positions described in the guideline are the ‘bi-axillary’ and ‘anterior-posterior’ position. The latter can be achieved by placing one pad in the standard apical position and the other on the right upper back or by placing one pad anteriorly over the left precordium and the other posteriorly to the heart just inferior to the left scapula.^2^

Given the importance of effective defibrillation, correct teaching of this skill is crucial. The relevance of this is underscored by Heames, Sado and Deakin 2001^5^ who analysed 101 sets of sternal-apical defibrillator pads placed by ALS *providers*, finding that only 22% of the apical pads were positioned correctly, with the majority placed too medial and too cranial.^5^ Difficulty with pad placement is not limited to adults. Also in (older) children only 26% of pads were found to be placed correctly.^6^ Studies on ALS instructors performance of defibrillation are currently lacking. Given the importance of correct defibrillation and the current interest in alternative defibrillation positions,^7^, ^8^, ^9^, ^10^ we analysed ALS-certified instructors’ performance of defibrillation pads placements.

## Methods

### Study design

This study used an observational simulation-based cohort and described according to the extended CONSORT and STROBE guideline for simulation-based research.^11^

### Setting

This research was conducted at a large ALS training institute in the Netherlands. Instructors were asked to participate in the research study which was done on site. Data were collected between May and October 2024.

### Participants

A total of 50 ERC-certified ALS instructors were recruited from the training institute, all instructors participated individually and voluntarily.

### Outcome parameters and measurements

Correct defibrillation pad position was defined as primary outcome. Secondary outcomes were performance self-assessment, defibrillation experience, self-perceived competence and self-efficacy in teaching defibrillation.

Correct defibrillation pad positions were determined by two experts, being (interventional) cardiologists with over 20 years of clinical experience and well established teaching experience as course directors for the ALS (re)certification courses. Each expert placed four defibrillation pads in the sternal, lateral, anterior and posterior position individually and according to the contemporary ALS guideline twice, at least one week apart. Centre points and angles of the defibrillation pads were recorded in reference to permanent markings on the mannikin and averaged to four reference positions.

Centre points of pads placed by the participants within a 50 mm diameter and within a 45 degree angle in regards to the reference positions were marked as correct, centre points and angles outside these ranges were marked as incorrect.

### Equipment

An anatomically correct rescue dummy (CrashTest-Service® GmbH, Germany) from the Fire and Rescue Department was used to resemble a real size and weight-based human body. The length of the mannikin was 172 cm with an abdominal and chest circumference of 99 and 102 cm respectively and a total body weight of 90 kg. Participants placed two sets of disposable, self-adhesive pads, sized 88x132mm, with unrestricted cable length and without time constraints.

### Survey

After pad placements, participants completed an online survey consisting of five demographic questions, four questions regarding personal experience with (alternative) defibrillation positions (six-point experience scale) and 11 self-reported efficacy/competence questions on 11-point Likert scales ranging from “strongly disagree” (0) to “strongly agree” (10). The survey was made in Qualtrics’ online research platform (Qualtrics™, Provo, UT, USA) and linked to pad placements through a unique identifier provided by the researcher.

### Bias

Measurement bias is minimized by the creation of standardized measurement forms with predefined distances and positive/negative deflections and one dedicated researcher (DW) performing all measurements. All pad positions where photographed and measurements verified by a second researcher (FC). Researcher bias is further limited as the data analysis was performed by a third researcher (LM).

### Statistical analysis

Data were analysed using IBM Statistic Package for the Social Sciences (IBM Corp.© SPSS Statistics for Windows, Version 28.0.0.0). Dichotomous variables (correctness of pad position) were reported as counts and percentages. Ordinal and scale variables were reported as mean ± standard deviation. Associations between non-parametric variables were analysed using Spearman’s rank-order correlation test. Internal consistency between the self-efficacy teaching defibrillation was analysed using Cronbach’s Alpha.

### Ethics

The study was reviewed by the Institutional Review Board of METC East Netherlands of the Radboud University Nijmegen Medical Centre (2024-17263). On this basis, the METC East Netherlands declared that this research does not fall under the WMO. This study was carried out in accordance to the applicable legislation. All participants were informed of the study prior to data collection and had an opportunity to ask additional questions before they provided informed consent. All data were collected anonymously.

## Results

A total of 50 instructors participated in the study, 24 males, 26 females with the majority (54%) being medical doctor either as consultant (46%) or as resident (8%). Registered nurses made up 38% of the participants. Participants’ demographics are shown in Table 1. Participants had a mean age (SD) of 42.1 (9.96) years. The majority of instructors were working in emergency medicine (34%) and intensive care medicine (28%).

**Table 1:** Participants’ demographics (*N* = 50).

**Sex**	
Male	24 (48%)
Female	26 (52%)
**Age**	
Mean (SD) yrs	42.1 (9.96)
**Background**	
Consultant	23 (46%)
Registered nurse	19 (38%)
Resident	4 (8%)
Physician assistant	4 (8%)
**Medical profession**	
Emergency Medicine	17 (34%)
Intensive Care Medicine	14 (28%)
Anaesthesia	7 (14%)
Cardiology/coronary care	6 (12%)
Ambulance service	3 (6%)
Other*	3 (6%)
**Instructors’ teaching experience**	
0–5 years	22 (44%)
6–10 years	15 (30%)
11–15 years	8 (16%)
16–20 years	4 (8%)
>20 years	1 (2%)

Values represent numbers (N) and percentages (%) unless specified otherwise.

* Includes: surgery and ‘not specified’.

Applied defibrillator pads had a mean ± SD distance to its corresponding reference point of 42 ± 21 mm, 38 ± 23 mm, 35 ± 19 mm and 61 ± 48 mm for the sternal, apical, anterior and posterior pads respectively. Of all defibrillation pads applied (*N* = 50 per position), 18% of the sternal pads, 20% of the apical pads, 32% of the anterior pads and 28% of the posterior pads were placed correctly as shown in Table 2. The majority of incorrectly placed sternal pads were placed too cranial (62%) and too lateral (68%). Incorrect apical pads were placed too cranial in 34%, too caudal in 34%, too posterior in 28% and too anterior in 42%. Only one respondent placed both sternal and apical pads correctly. Of all the apical pads, 21 (42%) were positioned excessively anteriorly. Among these, two cases also exhibited medially misplaced accompanying sternal pads, potentially resulting in a more anteriorly directed vector current.

**Table 2:** Position and correctness of defibrillation pad positions (*N* = 50 per position).

	Sternal pad	Apical pad	Anterior pad	Posterior pad
**Distance from reference (mm)**				
Mean	42	38	35	61
SD	21	23	19	48
Minimum	1	8	5	5
Maximum	83	144	99	190
**Correct overall (N, %)**	9 (18%)	10 (20%)	16 (32%)	14 (28%)
**Incorrect centre point (N, %)**	36 (72%)	35 (70%)	34 (68%)	36 (72%)
Too cranial (N, %)	31 (62%)	17 (34%)	14 (28%)	6 (12%)
Too caudal (N, %)	5 (10%)	17 (34%)	20 (40%)	30 (60%)
Too lateral / posterior (N, %)	34 (68%)	14 (28%)	25 (50%)	15 (30%)
Too medial / anterior (N, %)	2 (4%)	21 (42%)	9 (18%)	21 (42%)
**Incorrect angle (N, %)**	11 (22%)	17 (34%)	0 (0%)	0 (0%)

**Fig. 1:**
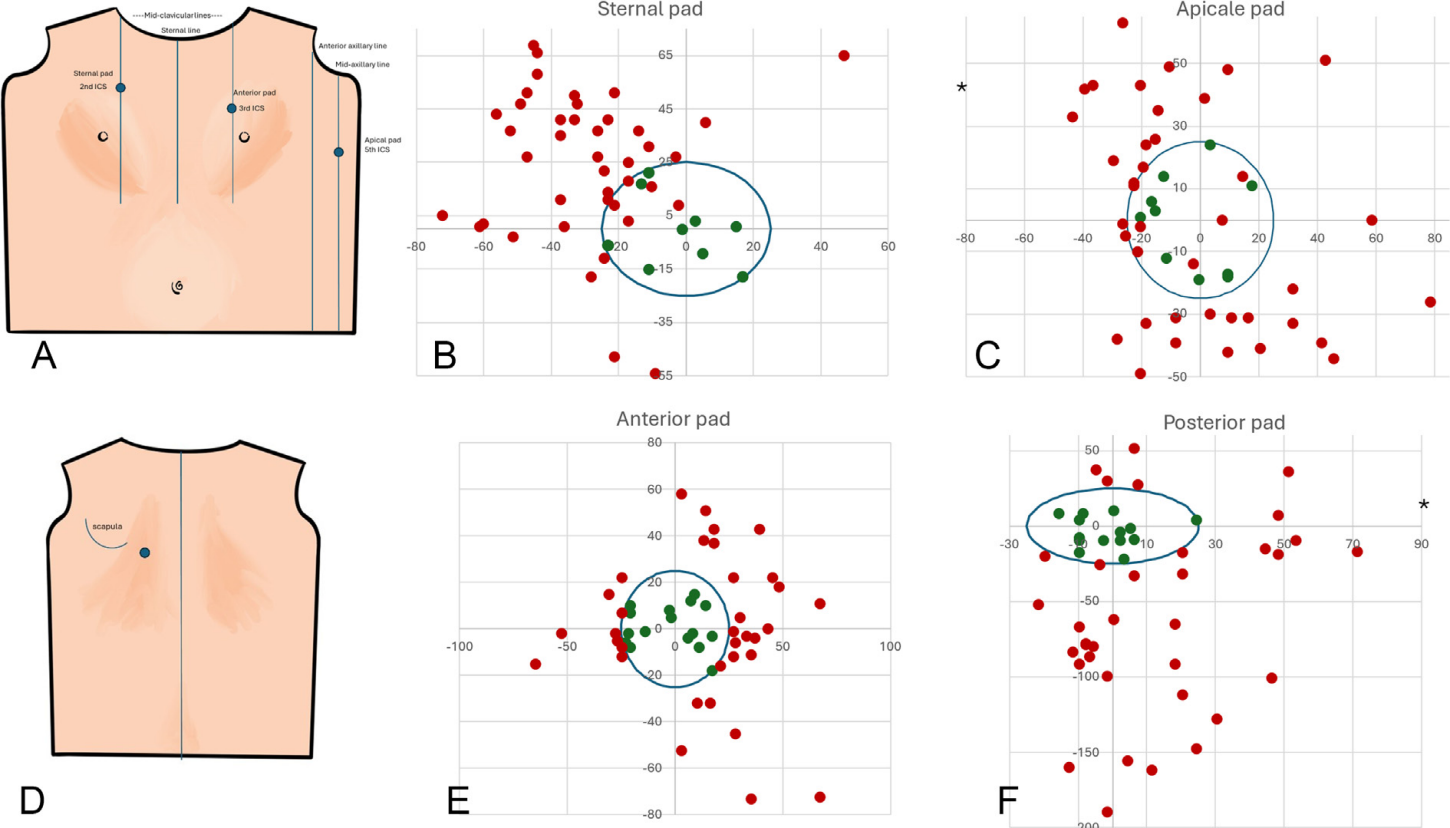
Individual pad placements. Panels A and D show schematic representations of defibrillation pads according to contemporary resuscitation guideline. Panels B, C, E and F represent pad placements (50 per position). Grid axes represent distances (mm) from correct position as determined by experts. Blue circles represent 50mm diameters. Green dots represent correctly placed pads. Red dots represent incorrectly placed pads. Abbreviations: ICS = intercostal space, * value outside range. (For interpretation of the references to colour in this figure legend, the reader is referred to the web version of this article).

Incorrectly positioned anterior pads were predominantly located too laterally (*N* = 25, 50%) and too caudally (*N* = 20, 40%) relative to the designated reference point. Posterior pads with improper placement were primarily situated too caudally (*N* = 30, 60%) and too medially (*N* = 26, 42%), while 30% were positioned too laterally and only 12% too cranially, as illustrated in Fig. 1. Only two respondents placed both anterior and posterior pads correctly. In 30% (*N* = 15) of all anterior-posterior sets, *both* pads were either placed too caudal (*N* = 12) or too cranial (*N* = 3), whereas in 26% (*N* = 13), both pads were either placed too lateral (*N* = 9) or too medial (*N* = 4), thereby altering the current vector accordingly.

The average number ± SD of correctly applied pads per instructor was 0.98 ± 0.74 out of four, with a minimum of 0 and a maximum of 3.

Self-assessment of pad placements for sternal-apical and anterior-posterior pads was 8.56 ± 1.33 and 7.88 ± 1.64 respectively. A Spearman's rank-order correlation was performed to examine the relationship between self-assessment and actual performance. No significant correlation was found between the two variables (rs(50) = 0.17, *p* = 0.241 and rs(50) = 0.12, *p* = 0.416 for the sternal-apical and anterior-posterior positions respectively).

**Fig. 2:**
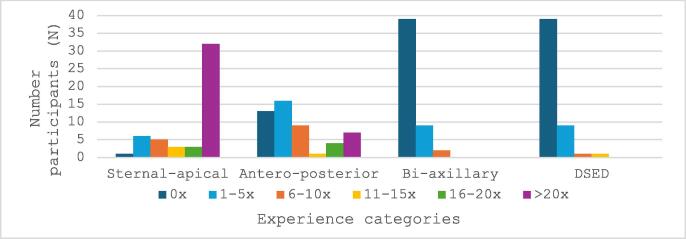
Participant’s experience with different defibrillation positions (*N* = 50 participants).

When analysing participants’ experience with actual defibrillator pad placements, the majority of participants (*N* = 32, 64%) had applied over 20 standard (sternal-apical) defibrillations. Experience with anterior- posterior pad placements was more heterogenous: 26% had no experience, 32% had 1–5 experiences, 18% had 6–10 experiences and 24% reported >10 real-life experiences. Experience with bi-axillary and double sequential external defibrillation (DSED) were absent in 78% of participants as displayed in Fig. 2. Spearman’s rank-order correlation showed no association between experience and actual performance (rs(50) = 0.11, *p* = 0.463 and rs(50) = −0.16, *p* = 0.256 for sternal-apical and anterior-posterior sets respectively).

Self-perceived competence scores (mean ± SD, Likert scale 0 to 10) amongst instructors in teaching sternal-apical, anterior-posterior, bi-axillary and DSED positions were 8.68 ± 1.06, 8.08 ± 1.37, 5.57 ± 2.95 and 5.11 ± 2.67 respectively, see Table 3.

**Table 3:** Self-reported competence and efficacy on teaching defibrillation (*N* = 50 participants).

	Min	Max	Range	Mean	SD
**Self-reported competence in teaching correct:**					
Sternal-apical positions	6	10	4	8.68	1.06
Anterior-posterior positions	4	10	6	8.08	1.37
Bi-axillary positions	0	10	10	5.57	2.95
Dual Synchronized External Defibrillation positions	0	10	10	5.11	2.67
**Self-efficacy ‘teaching defibrillation’ (construct)**					
I feel confident teaching the correct standard and alternative defibrillation pad positions	5	10	5	8.11	1.17
I am capable explaining the working and settings of the manual and/or automatic external defibrillator	6	10	4	8.70	1.06
I am capable of discussing relevant safety aspects and potential complications of defibrillation	6	10	4	8.62	1.03
I am capable of answering question regarding defibrillation in general and in more specific circumstances	6	10	4	8.47	0.86
I feel confident explaining the differences between (asynchronous) defibrillation and (synchronous) cardioversion	6	10	4	9.04	1.00

A Spearman's rank-order correlation was performed to examine the relationship between self-perceived competence and actual performance. No significant correlation was found between the two variables (rs(50) = 0.12, *p* = 0.401 and rs(50) = −0.12, *p* = 0.416 for the sternal-apical and anterior-posterior positions respectively).

Self-efficacy score for teaching defibrillation was 8.59 ± 0.81. This construct was made up of five questions, with a Cronbach’s Alpha of 0.845, indicating a high internal consistency and reliability. Self-efficacy was not related to the number of correctly applied pads (Spearman’s rank-order rs(47) = 0.02, *p* = 0.880).

## Discussion

This study provides compelling evidence that defibrillator pad placement remains a challenging task, corroborating and expanding upon existing knowledge. While previous research established the difficulty of pad placement, our study is the first to incorporate detailed analyses of pad orientations as well as anterior-posterior positions.

This study uniquely quantifies the extent of incorrect pad placement, demonstrating that only one-fifth to one-third of all defibrillation pads were positioned correctly by instructors. Despite the attention for longitudinal placement of especially the apical pad, our findings reveal that 22% of sternal pads and 34% of apical pads are placed in a more transverse orientation. Notably, we found no significant association between pad placement accuracy and factors such as self-evaluation, personal experience, perceived competence or self-efficacy in teaching defibrillation. These novel aspects offer further insight in the complexity of (teaching) defibrillator pad placement.

Difficulty achieving correct placement of external defibrillation pads is a known phenomenon, involving both laypersons^12^, ^13^ as well as health care professionals.^5^, ^6^, ^14^ Nurmi, Rosenberg and Castren 2004^14^ found that only 25% of health care professionals placed both sternal-apical pads correctly. Heames, Sado and Deakin 2001^5^ found 65% of sternal pads and 22% of apical pads being correctly applied. In our study these percentages were even lower. The lower success rates are partially explained by incorporating pad orientation which previous studies did not. As an example: the ‘correct’ placement by Nurmi, Rosenberg and Castren 2004^14^ is regarded incorrect in our study since the apical pad is placed in a transverse rather than a longitudinal direction.^15^ Even when pad orientation is omitted from the analyses, only 28% and 30% of sternal and apical pads respectively were placed correct in our study. Especially the sternal pad shows lower success rates in our study, which might relate to the description of the correct place, allowing multiple interpretations: “the sternal pad is placed to the right of the sternum, below the clavicle”.^2^ Also instructional pictograms are often different and open to multiple interpretations.^13^, ^16^

Implications of misplaced defibrillation pads are difficult to quantify since studies correlating (biphasic) defibrillation pad placement in relation to defibrillation success are lacking.^17^ Although different pad positions are shown to result in similar transthoracic impedances,^18^, ^19^ the vector and/or available current through the myocardium can possibly change.^20^, ^21^ Our study highlights this importance with over a third of all sets (sternal-apical and anterior-posterior) most likely resulting in a vector deviation. Although the sternal pad position had the highest misplacements, it probably has the smallest effect on the vector change. Theoretical modelling showed however that a 25–50 mm displacement of the anterior pad results in a 4–14% current change.^22^ The definitions of correct placement in our study were arbitrarily set to a 50 mm diameter and 45 degree angle. This distance was used in previous studies^5^, ^14^ and initially derived from being the radius of a defibrillation paddle. The angle cut-off was derived from the study by Deakin, Sado, Petley and Clewlow 2003^15^ who found a reduced impedance of the apical paddle when placed in a longitudinal direction compared to the transverse position.

Clinical studies on the implications of defibrillator pads (mis)placement are still limited, making it difficult to determine the ‘gold standard’ for correct defibrillation pad positions. The contemporary resuscitation guidelines have determined their pad recommendations from the best available evidence. The correct position of defibrillation pads in our study was determined by two experts according to the resuscitation guideline. By using a dual-expert approach with repeated measurements we strived for optimal precision. The variance amongst instructors’ performance raises concerns on current defibrillation practices.^23^ Research has highlighted the critical role of instructors’ competence in teaching, yet their performance shows substantial variation. Also, instructors’ skills evaluation is known to vary and individual skill retention over time is poor, necessitating frequent training.^23^, ^24^, ^25^ Innovative approaches such as augmented reality could perhaps be beneficial in improving or retaining proficiency through the provision of more objective feedback.^25^, ^26^, ^27^ In addition, more detailed descriptions and uniform visual representations could further help standardize defibrillator pad placements.^13^, ^28^ By integrating these recommendations, we can work towards more consistent and effective defibrillator pad placement, potentially leading to improved patient outcomes.

## Limitations

The determination of correct pad placement in this study relied on expert opinion based on the current resuscitation guideline, which may introduce a degree of subjectivity. The scarcity of clinical studies relating defibrillator pad placement to patient outcome limits our ability to establish a definitive 'gold standard' for accurate positioning. The composition of the human body (e.g. breast tissue) should be taken into account when placing defibrillation pads.^29^ The results from this study might therefore vary depending on body composition and dimensions. Furthermore, application of defibrillation pads during real resuscitation events is much more difficult due to the urgency, perceived stress, patients’ clothing, etcetera. With a primary focus on ‘knowledge’ in this study, we purposefully excluded these variables and let the instructors apply the pads without time constraints on an unclothed mannikin. Application of defibrillation pads under operational circumstances can therefore show even higher variances; a study that is currently being undertaken.

## Conclusion

This study corroborates and expands upon existing knowledge regarding the accuracy of defibrillator pad placement, revealing substantial variation in placement accuracy among instructors. Our analysis of pad orientation and anterior-posterior analysis demonstrates that a significant portion of pads are incorrectly placed. These findings highlight the need for standardized approaches and improved training methodologies in defibrillator pad placement.

## CRediT authorship contribution statement

**Dennie Wulterkens:** Investigation. **Freek Coumou:** Data curation. **Cornelis Slagt:** Writing – review & editing, Conceptualization. **Reinier A. Waalewijn:** Writing – review & editing. **Lars Mommers:** Writing – original draft, Formal analysis, Conceptualization.

## Funding

This research did not receive any specific grant from funding agencies in the public, commercial, or not-for-profit sectors.

## Declaration of competing interest

The authors declare that they have no known competing financial interests or personal relationships that could have appeared to influence the work reported in this paper.
