# Supplementary material for: Defibrillation pad placement accuracy among Advanced Life Support instructors: A manikin-based observational study examining experience, self-evaluation, and actual performance
Source: Resusc Plus. 2025 Feb 1;22:100886. doi: 10.1016/j.resplu.2025.100886 (PMC11851189; doi:10.1016/j.resplu.2025.100886)
Supplement: Supplementary Data 1 [file mmc1.docx]

**Online supplement**

Suppl1. Experience with (alternative) defibrillation positions amongst participants (N=50)

|  | **Defibrillation position** | | | |
| --- | --- | --- | --- | --- |
| **Experience** | Sternal-apical | Antero-posterior | Bi-axillary | DSED |
| Never | 1 (2%) | 13 (26%) | 39 (78%) | 39 (78%) |
| 1-5x | 6 (12%) | 16 (32%) | 9 (18%) | 9 (18%) |
| 6-10x | 5 (10%) | 9 (18%) | 2 (4%) | 1 (2%) |
| 11-15x | 3 (6%) | 1 (2%) | 0 (0%) | 1 (2%) |
| 16-20x | 3 (6%) | 4 (8%) | 0 (0%) | 0 (0%) |
| >20x | 32 (64%) | 7 (14%) | 0 (0%) | 0 (0%) |
